# Geotechnical Seismic Isolation System Based on Rubber-Sand Mixtures for Rural Residence Buildings: Shaking Table Test

**DOI:** 10.3390/ma15217724

**Published:** 2022-11-02

**Authors:** Zhiyong Yin, Haifeng Sun, Liping Jing, Rui Dong

**Affiliations:** 1 School of Civil and Architecture Engineering, Hunan University of Arts and Science, Changde 415000, China; 2 Key Laboratory of Earthquake Engineering and Engineering Vibration, Institute of Engineering Mechanics, China Earthquake Administration, Harbin 150080, China; 3 Key Laboratory of Earthquake Disaster Mitigation, Ministry of Emergency Management, Harbin 150080, China; 4 Zhuhai Engineering Investigation Institute of Guangdong Province, Zhuhai 519000, China

**Keywords:** rubber, base isolation, geotechnical seismic isolation, shaking table test, rural buildings

## Abstract

The anti-seismic problem of rural residential buildings is the weak link of seismic retrofitting in China. Recently, geotechnical seismic isolation (GSI) technology based on rubber–sand mixtures (GSI–RSM) using rubber–sand mixtures (RSM) between the structural foundation and the foundation soil has been proven to have the possibility of potential applications in rural residential buildings. Many theoretical studies exist on the effectiveness of seismic isolation of the GSI–RSM system, but few studies on either the seismic response test of model buildings placed on the RSM layer or the large-scale shaking table test exist. Therefore, this study considers a large shaking table test performed on a 1/4 single-story masonry structure model with and without a GSI–RSM system by selecting a standard input ground motion and varying input acceleration amplitudes. The test results show that the GSI–RSM system can reduce the seismic response of superstructures. The isolation effect of the GSI–RSM system is low in small earthquakes and increases with increasing earthquake magnitude. Overall, the RSM layer can filter part of the high-frequency components of the earthquake to transmit to the superstructure and consume more seismic energy by generating friction slip in the interaction with the structural foundation.

## 1. Introduction

The Wenchuan and Yushu earthquakes of 12 May 2008 and 14 April 2010, respectively, caused numerous casualties and huge economic losses in rural China. Masonry structures are some of the largest residential structural forms of residences in rural areas of China. Due to the lack of appropriate seismic fortification measures for masonry structures in rural areas, the disasters caused by masonry structures are very critical and often have the characteristics of numerous collapsed houses, a wide disaster area with a dispersed disaster population and difficult rescue, etc. For developing countries, it is imperative to study the seismic and damping problems of rural residential buildings.

Generally, there are two main ways of reducing earthquake disasters in buildings, namely, seismic reinforcement and isolation technology. Currently, isolation technology is relatively developed and widely applied. Among the two methods of earthquake disaster reduction, base isolation technology is where rubber isolation bearings are set between the foundation and the superstructure [1,2,3,4]. However, base isolation technology is expensive and can only be seen in major and expensive structures; therefore, it is unsuitable for popularization and application in constructing rural residential buildings. Recently, there have been some new concepts and developments in isolation systems and devices. Geotechnical seismic isolation (GSI) technology using low modulus materials around the structural foundation has aroused extensive research interest. Tsang [5] first proposed the GSI technology, which uses rubber–sand mixtures (RSM) as foundation pit backfilling material. The isolation effectiveness of the geotechnical seismic isolation based on the rubber–sand mixture (GSI–RSM) system was confirmed through several numerical simulations and parameter research. Due to the increasing number of vehicles on the road, waste tire disposal has become a serious environmental problem in many cities. The GSI–RSM system is a sustainable and environmentally friendly [6] technology because it uses many crushed waste tires as rubber materials, which helps to reduce waste tire accumulation. Concurrently, the GSI–RSM system is inexpensive; therefore, it is appropriate for popularization and application in developing countries where resources and technology are insufficient to use developed but expensive technology to mitigate earthquakes.

Although the GSI concept has been around for some time and is promising regarding environmental protection and recycling, as far as we know, it has not yet been applied in practical engineering projects. Nevertheless, people are still trying to prove their effects mainly through the following two methods.

On the one hand, researchers have performed several numerical simulation works. For example, Senetakis et al. [7] and Pitilakis et al. [8] studied the response reduction of a single-degree of freedom pendulum model. The isolation effect of GSI on residential buildings has been extensively studied, and all results showed that GSI can reduce the seismic response of buildings [9,10,11,12,13]. The results of Dutta et al. [14] showed that waste rubber–soil mats can reduce the seismic liquefaction risk of structures. In addition, Forcellini and Alzabeebee [15] investigated the seismic vulnerability of GSI to bridge structures. More recently, Brunet et al. [16] introduced a nonlinear inelastic model into the numerical simulation of the GSI–RSM system for building structures to quantify the reduction of seismic demand caused by RSM layers. Tsang and Pitilakis [17] developed lumped parameter analysis models for GSI system dynamic analysis of buildings, in which the authors put forth equivalent linear stiffness and viscous damping coefficients, alongside oscillating radiation damping coefficients of ground and embedded foundations.

On the other hand, many experimental studies were carried out. For example, Hazarika [18] and Hazarika et al. [19] conducted model shaking table tests on caisson wharf walls and underground pipelines with RSM as vibration isolation backfilling, which showed that RSM vibration isolation backfilling technology greatly reduced seismic force and reduced residual displacement and liquefaction. Kaneko et al. [20] conducted a pseudo-dynamic response test, and the test results showed that the GSI system is effective in seismic isolation and liquefaction prevention. Xiong and Li [21] and Bandyopadhyay [22] placed RSM as isolation material under concrete or rigid block for a small shaking table test, which verified the isolation effectiveness of the GSI–RSM system. Anastasios et al. [23] studied the mechanical properties of the potential failure mechanism inside the RSM layer through the direct shear test, which further confirmed its applicability to seismic isolation. Tsang et al. [24] firstly examined the performance of the GSI–RSM system in a geotechnical centrifuge and found that an average of 40–50% reduction of structural demand can be achieved. Pitilakis et al. [25] conducted a large-scale experimental study on a prototype structure to assess the effectiveness of gravel–rubber mixture (GRM) layers underneath shallow foundations as a means of GSI.

In the past decade, many numerical simulations and experimental studies have been performed on the effectiveness of seismic isolation of the GSI–RSM system, which has proven that the GSI–RSM system has potential application possibilities in rural residential buildings. However, there are few reports on the seismic response test of model buildings placed on the RSM layer, and as far as we know, large-scale shaking table tests have not been done. More research needs to be conducted in large-scale laboratory or field tests before the GSI–RSM system can be used in practical engineering. Therefore, in this study, we aimed to carry out large-scale shaking table tests based on the GSI–RSM system considering the scale model of a single-layer masonry structure on the site, verify the effectiveness of the GSI–RSM system, and discuss the isolation mechanism of the GSI–RSM system.

## 2. Shake Table Test

**Figure 1:**
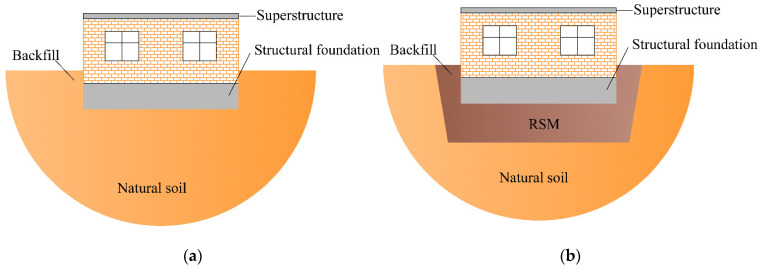
Schematic diagram of the GSI–RSM system: (**a**) without GSI–RSM system; (**b**) with GSI–RSM system.

Figure 1 shows the schematic diagram of rural residential buildings with and without the GSI–RSM system. The structural part in Figure 1 is a one-story masonry structure in rural areas of China, and the masonry structure is a typical structural form in rural residential houses. It can be seen from Figure 1a that the foundation of rural residential buildings without a GSI–RSM system is only a natural soil layer, while in Figure 1b, the foundation soil of rural residential buildings with a GSI–RSM system is composed of natural soil and an RSM layer. To study the isolation effect of the GSI–RSM system on rural residential buildings through the shaking table test, the shaking table tests in this paper included two groups. One was the rural residential buildings test without the GSI–RSM system, and the other was the rural residential test with the GSI–RSM system (hereinafter referred to as the non-isolation test and GSI–RSM test).

### 2.1. Test Equipment

**Figure 2:**
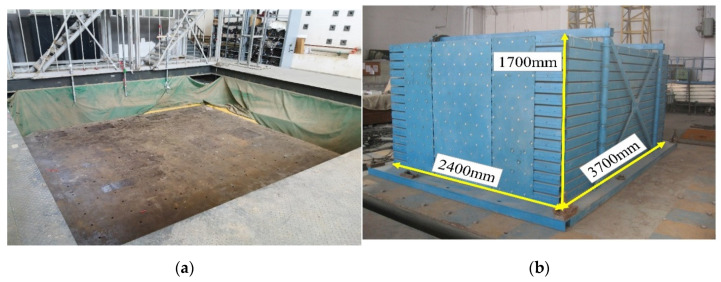
Test equipment: (**a**) shaking table system; (**b**) laminated shear box.

The shaking table test model included two parts, namely, the structural model and the foundation soil model, and the major equipment for this test was the shaking table system and a laminated shear box [26] (see Figure 2). The shaking table system had 6 degrees of freedom, and the table size was 5 m × 5 m. The maximum allowable bearing capacity of the table was 300 kN. The main body size of the laminated shear box was 3700 mm × 2400 mm × 1700 mm, and the base size was 4180 mm × 2820 mm × 120 mm. It was composed of 15 layers of a mouth-shaped steel tube frame. Each layer of the steel frame was welded by four mouth-shaped steel tubes. The section size of the mouth-shaped steel tube was 100 mm × 100 mm, and the wall thickness was 3 mm.

### 2.2. Model Similarity

The length, elastic modulus, and density were selected as the three basic quantities in the model similarity. Nevertheless, due to the size limitation of the shaking table and laminated shear box, the length similarity ratio was 1/4. Regarding the bearing capacity limitation of the shaking table and other test conditions, this test adopted the under-artificial mass model, that is, an artificial counterweight set on the structural model to supplement the deficiencies of the gravity and inertial effects. Furthermore, due to the particularity of soil material, it was difficult to simulate the gravity similarity condition of soil under constant acceleration in the shaking table test. Therefore, the similarity relationship of soil was not considered in the shaking table test, and the under-artificial mass model was used in the structural model to satisfy the similarity law of structural seismic response. Overall, the similarity of the main physical quantities in the structural model is shown in Table 1.

### 2.3. Test Model

#### 2.3.1. Structural Model

**Figure 3:**
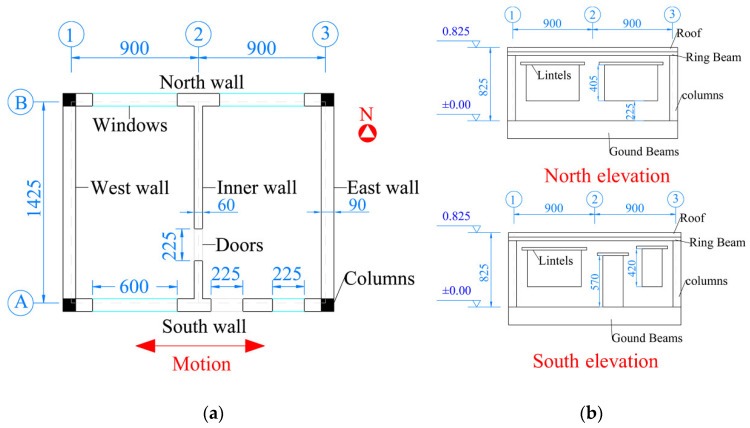
Plan and elevation of the structural model (unit: mm): (**a**) plan view; (**b**) elevation view.

A common kind of masonry structure in rural areas of China with structural columns, ring beams, and other seismic measures was selected as the prototype structure. The plane size was 7200 mm × 5700 mm, and the total height of the house was 3300 mm. According to the length similarity relationship, the plane size of the structure model was 1800 mm × 1425 mm, and the total height was 825 mm. The structural model plane and elevation are shown in Figure 3, and the vibration direction was east–west. The structural model mainly included a brick wall, structural column, ring beam, roof plate, and foundation beam. The plane size of the roof was 1920 mm × 1545 mm, and the thickness was 50 mm.

#### 2.3.2. Foundation Soil Model

**Figure 4:**
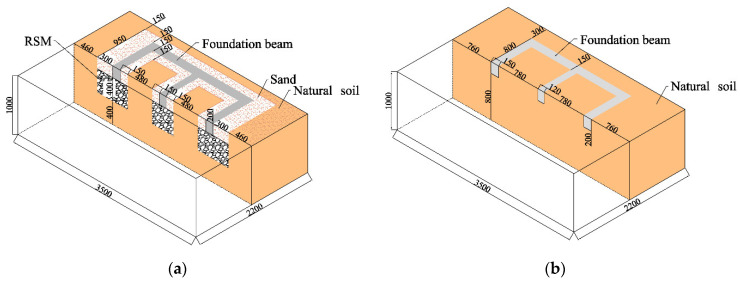
Model of foundation soil (unit: mm): (**a**) the GSI–RSM test; (**b**) the non-isolation test.

The foundation soil model was completed in the soil box and prepared through the divided compaction method. The soil box was a laminated shear box. The foundation soil model of the GSI–RSM and non-isolation test is shown in Figure 4. The figure shows that the total thickness of the soil layer in the foundation soil model was 1000 mm, and the entire buried depth of the structural foundation was 200 mm. The foundation soil model of the non-isolation test was composed of natural soil, while the foundation soil model of the GSI–RSM test was comprised of natural soil, sand, and RSM. The non-isolation test foundation soil model produced a 1000 mm thick natural soil foundation according to the layered compaction method, involving layer compaction and scraping, where each layer of soil was 200 mm thick, and finally natural soil was backfilled around the foundation beam. The GSI–RSM test foundation soil model first produced a 1000 mm thick natural soil foundation, and then a foundation trench with a depth of 600 mm was excavated based on the natural soil foundation, and then a 400 mm thick RSM was uniformly laid in the foundation trench. Finally, the sand was backfilled around the foundation beam.

### 2.4. Sensor Arrangement

**Figure 5:**
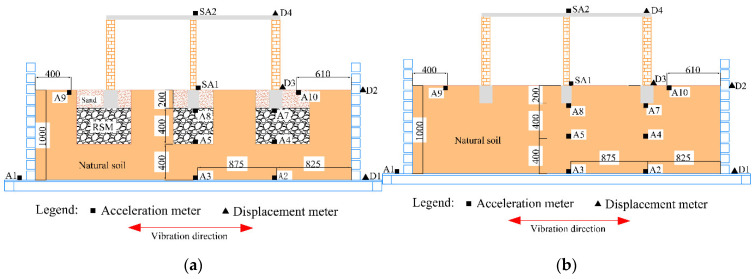
The arrangement of acceleration sensors and displacement sensors: (**a**) the GSI–RSM test; (**b**) the non-isolation test.

The acceleration and displacement sensors were mainly adopted in the shaking table test. The acceleration sensor was a piezoelectric acceleration sensor, and the displacement sensor was a wire-pull displacement meter. To obtain the dynamic response of the model, acceleration and displacement sensors were arranged on the shaking table, at different depths of the soil layer, on the structural model foundation and the roof panel. The arrangement of acceleration and displacement sensors in the GSI–RSM test and non-isolation test is shown in Figure 5. The acceleration sensor is represented by the letter A or SA to distribute acceleration sensors in soil layers and structural models, and the displacement sensor is represented by the letter D.

### 2.5. Seismic Wave and Loading System

**Figure 6:**
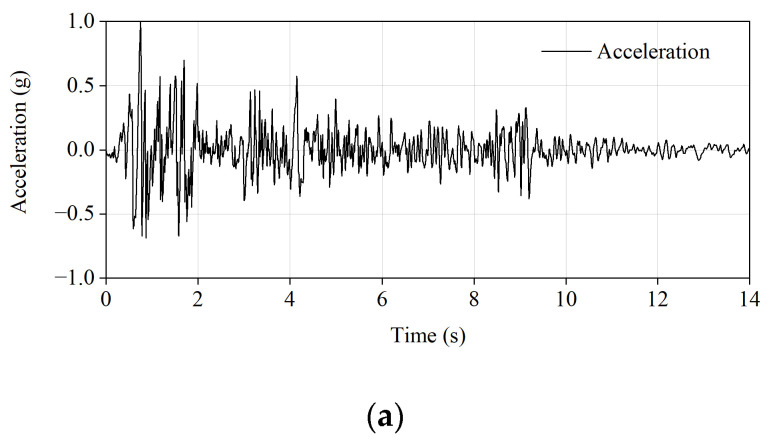

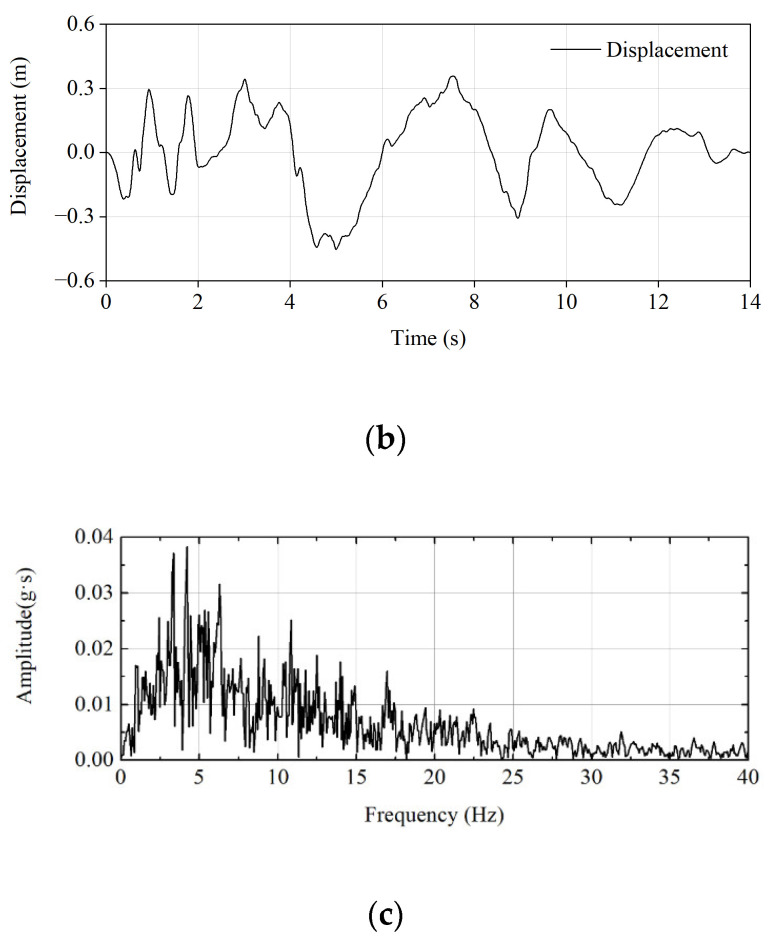
Characteristics of the input wave: (**a**) time history curve of acceleration; (**b**) time history curve of displacement; (**c**) acceleration Fourier spectrum curve.

The test was conducted in the Key Laboratory of Earthquake Engineering and Engineering Vibration, Institute of Engineering Mechanics, China Earthquake Administration. The site where the prototype structure was located was class II. According to the Code for Seismic Design of Buildings (GB50011-2010) [27], the north–south component of the EL Centro seismic wave recorded in California in 1940 was selected as the input ground motion, and horizontal (east–west) excitation was carried out on the model. The acceleration amplitude of the original EL Centro seismic wave (north–south component) was adjusted to 1.0 g, and the time-holding was compressed according to the time similarity relationship. The adjusted acceleration, displacement time–history curve, and Fourier spectrum of the input ground motion are shown in Figure 6. Before the test, white noise was inputted to sweep frequency, and then seismic waves with acceleration amplitudes of 0.1 g, 0.2 g, and 0.4 g were inputted in turn. The loading sequence of each ground motion intensity is shown in Table 2.

## 3. Materials and Methods

### 3.1. Structural Model

**Figure 7:**
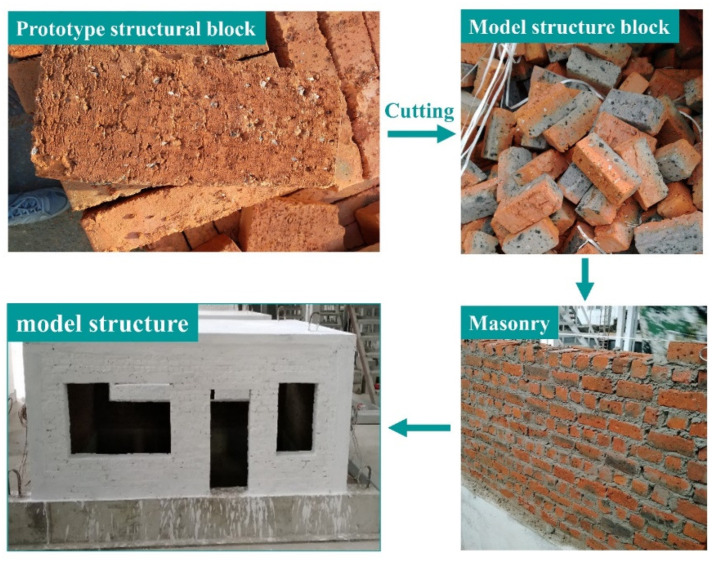
The production process of the structural model.

The block material of the prototype structure was a sintered ordinary brick with MU10 strength grade and 235 mm × 115 mm × 45 mm size. To make the structural model blocks using the same material as the prototype structure blocks, the lengths and widths of the bricks were cut according to the proportion of the length similarity ratio. Considering the limitation of construction conditions, the bricks were cut in the thickness direction according to the proportion of 1/2. After cutting, the brick size of the structural model measured 56 mm × 26 mm × 21 mm. The production process of the structural model is shown in Figure 7.

**Figure 8:**
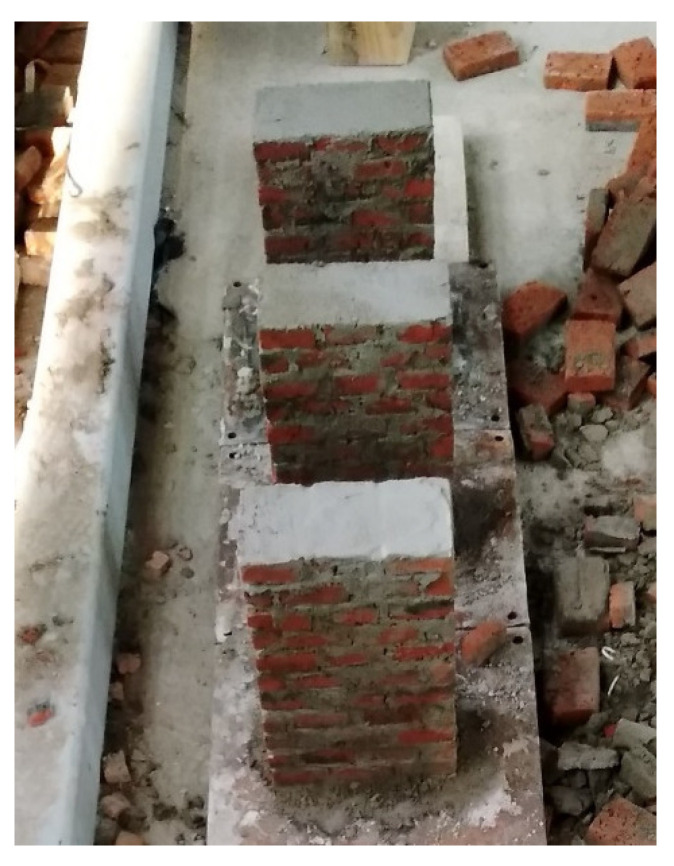
Masonry specimens.

In making the structural model, three masonry specimens were built (see Figure 8). The size of each specimen was 180 mm × 275 mm × 85 mm, and the height/thickness ratio was about 3.2. After curing, the elastic modulus test was carried out on the pressure testing machine. According to the standard test method for basic mechanical properties of masonry (GB/T50129-2011) [28], the secant modulus of the data point when the stress is 0.4 times the peak compressive stress was taken as the elastic modulus of the specimen. The compressive strength and elastic modulus of the masonry specimens are shown in Table 3.

### 3.2. Foundation Soil Model

**Figure 9:**
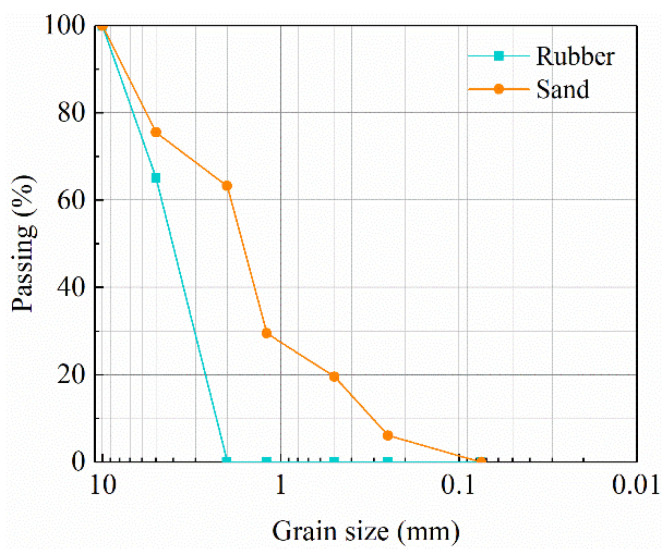
Cumulative gradation curve.

**Figure 10:**
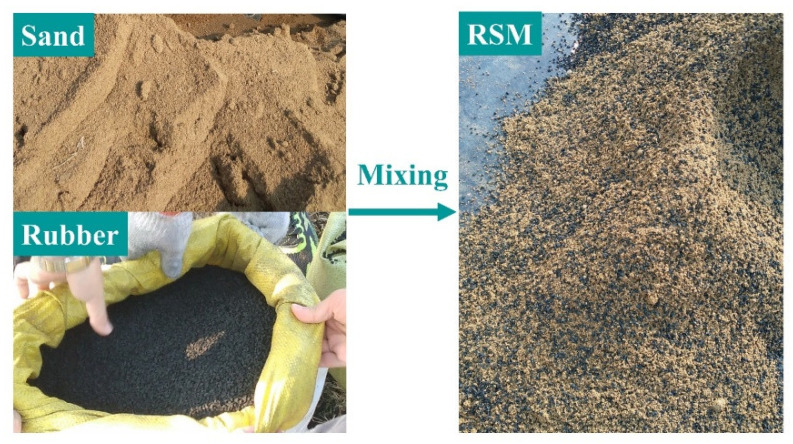
Mixing of rubber particles and sand.

**Figure 11:**
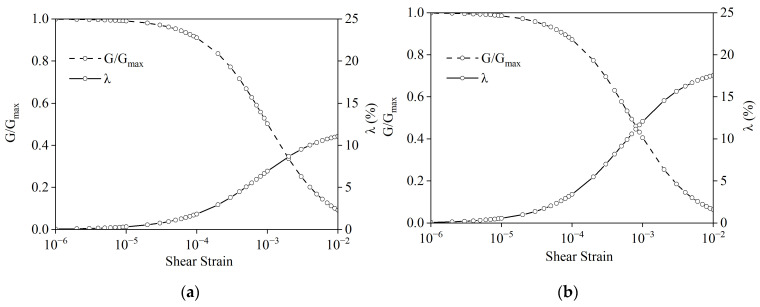
Curve of shear modulus ratio and damping ratio of soil with shear strain: (**a**) natural soil; (**b**) RSM.

The foundation soil model includes natural soil, sand soil, and waste tire rubber particles. The natural soil was selected as silty clay of a construction site, and the sand was selected as river sand. The particle size range of waste tire rubber particles was 3–6 mm. The gradation curves of sand and rubber particles are shown in Figure 9. The non-uniformity coefficient and curvature coefficient of sand were 10.37 and 0.0247, respectively, and the non-uniformity coefficient and curvature coefficient of rubber particles were 2.025 and 0.858, respectively. RSM was prepared by mixing rubber particles with sand according to volume (see Figure 10), where the volume ratio of rubber particles to sand was 1:4. The basic physical and mechanical parameters of natural soil and RSM are shown in Table 4. The curves of dynamic shear modulus ratio $G/G_{max}$ and damping ratio λ of natural soil and RSM with shear strain are shown in Figure 11.

## 4. Test Results and Discussion

### 4.1. Seismic Damage Analysis

#### 4.1.1. Structural Model

**Figure 12:**
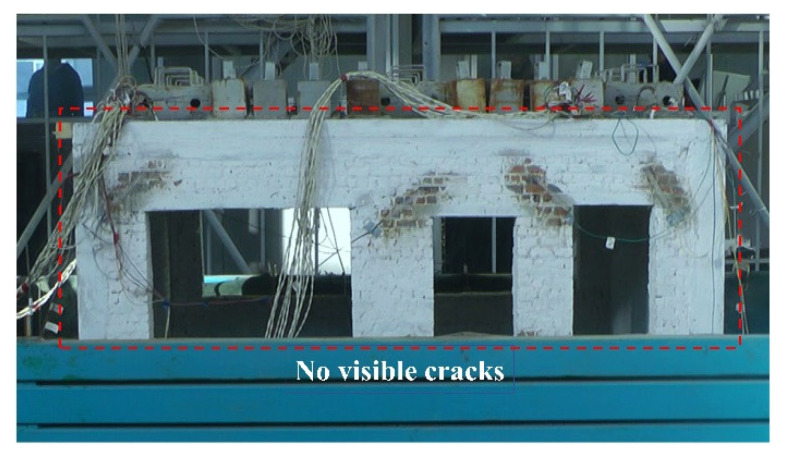
Cracks in the wall of the structure model of the GSI–RSM test.

**Figure 13:**
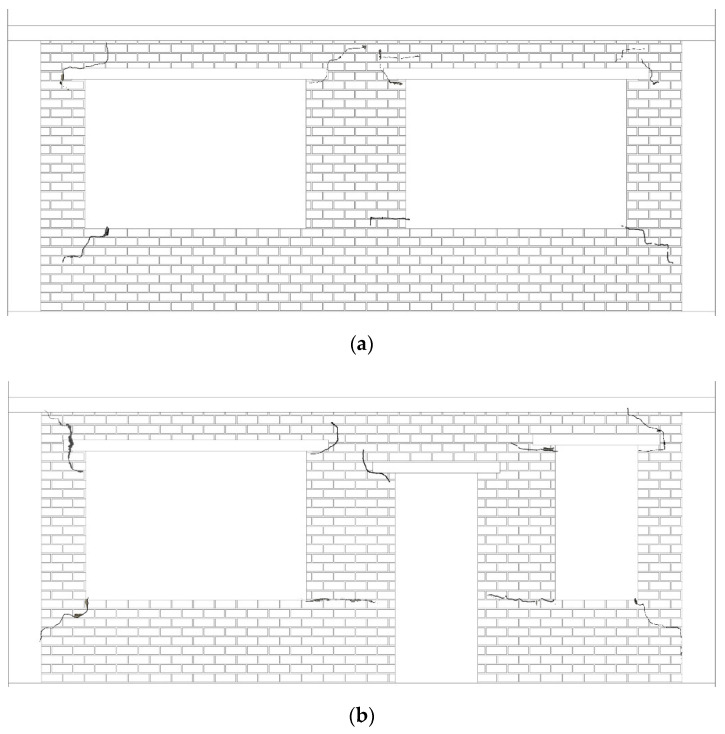
Cracks in the wall of the structure model of the non-isolation test: (**a**) north wall; (**b**) south wall.

Figure 12 shows the seismic damage of the structural model in the GSI–RSM test after loading. It can be seen that the structural model had no visible cracks. For the non-isolated test, no visible cracks were observed in the structure after the seismic loading with acceleration amplitudes of 0.1 g and 0.2 g. However, after the seismic loading with an input acceleration of 0.4 g, slight cracks appeared in the structure, and the complete crack distribution is shown in Figure 13. It can be seen that cracks mainly appeared in weak parts, such as doors and windows. Obviously, by comparing the macro seismic damage of the structural model in the non-isolation test and the GSI–RSM test, it can be seen that rural houses with a GSI–RSM system can effectively avoid the occurrence of brick wall cracks and reduce the structural damage of the superstructure.

#### 4.1.2. Foundation Soil Model

The most vulnerable part of the foundation soil model is the backfill soil near the structural foundation. During the test, the damage of backfill was the focus, and the settlement deformation of the structural model was measured at the beginning and end of the test.

**Figure 14:**
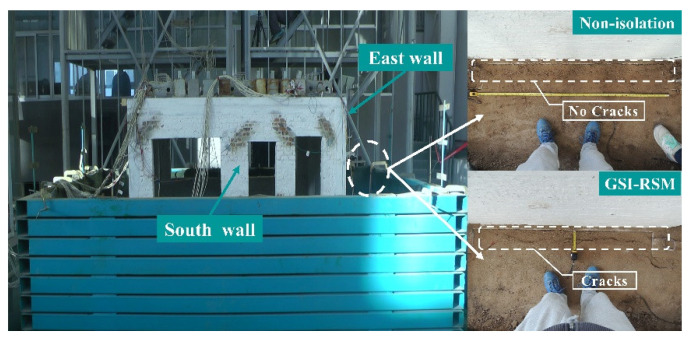
Backfill cracks of models.

During the test, when the acceleration amplitude of 0.1 g and 0.2 g seismic loading was completed, the backfill part of the two groups of tests did not produce significant damage. The acceleration amplitude of 0.4 g seismic load after the completion of the backfill damage is shown in Figure 14. Obviously, in the non-isolated test, after the whole seismic loading, there was no obvious damage to the backfill soil, indicating that there was no obvious relative slip motion between the structural foundation and the foundation soil in the non-isolated test. However, in the GSI–RSM test, cracks appeared in the backfill portion. According to the analysis of the causes, the relative slip motion between the structural foundation and the RSM layer was generated under the action of the horizontal earthquake, and the structural foundation and the backfilled sand collided and were squeezed by each other, gradually forming cracks.

**Figure 15:**
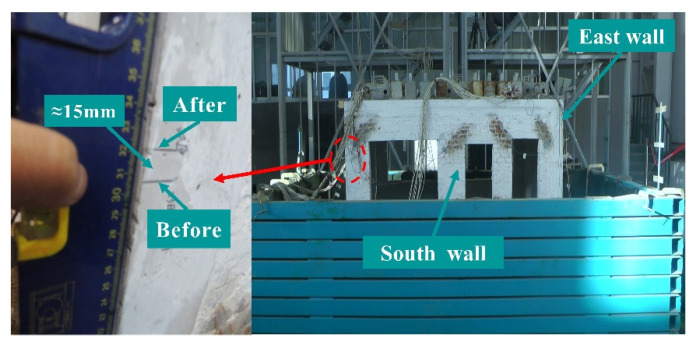
Settlement of models.

It is worth noting that after the completion of the seismic load loading, the position of the structural model of the GSI–RSM test was reduced by about 15 mm compared with that before the test (see Figure 15), while the position of the structural model in the non-isolation test remained unchanged. Comparing the composition of the foundation soil model in the non-isolation test and the GSI–RSM test, it can be inferred that the settlement of the structure model in the GSI–RSM test mainly came from the vertical deformation of the RSM layer. This is because under the excitation of increasing seismic amplitude, the RSM layer was squeezed and staggered between the particles, and the edges and corners of the sand particles were destroyed, resulting in the rolling of the particles and the rearrangement of the positions. Therefore, the pores of the RSM layer were reduced and gradually compacted by vibration. Finally, the total thickness of the RSM layer and the position of the structural model were reduced.

In summary, under the action of the earthquake, the damage deformation of the non-isolation test mainly occurred in the superstructure. However, the deformation of the GSI–RSM test mainly occurred in the RSM layer and backfill soil, and there was no obvious damage to the superstructure in the GSI–RSM test. It can be seen that the GSI–RSM system dissipated seismic energy mainly through the plastic deformation of the RSM layer and backfill sand and the relative slip motion between the structural foundation and RSM layer, so that the seismic energy transmitted to the superstructure was reduced.

### 4.2. Acceleration Response

#### 4.2.1. Fourier Spectrum

**Figure 16:**
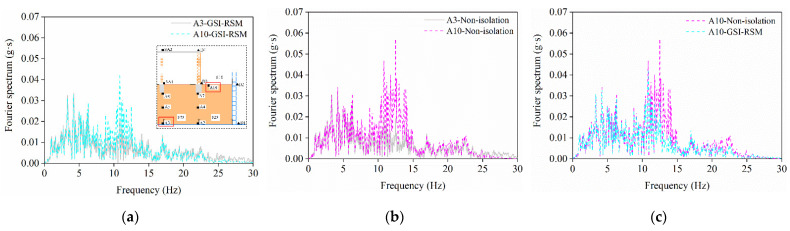
Fourier spectrum of measuring points: (**a**) GSI–RSM test; (**b**) non-isolation test; (**c**) comparison between non-isolation test and isolation test.

Figure 16 shows the Fourier spectra of measuring point A3 at the bottom of the soil layer and measuring point A10 on the soil surface under 0.1 g peak acceleration of the two test groups. It can be seen from the analysis that after the ground motion at the bottom of the soil layer passed through the foundation soil model, the Fourier spectrum of seismic motion at the soil surface measuring point A10 changed significantly. When the middle and high-frequency bands were 7–15 Hz and the high-frequency bands were 25–30 Hz, the spectrum value increased significantly, while the other frequency bands had little change, which showed that the medium-frequency and high-frequency components of foundation soil were amplified. Further analysis showed that compared with the non-isolation test, the 11–15 Hz spectral value of the GSI–RSM test in the high-frequency band was significantly reduced; that is, the RSM layer had an obvious filtering effect compared with the natural soil to suppress the high-frequency component. This indicates that the seismic response of masonry structures with GSI–RSM system under seismic loading will be reduced.

#### 4.2.2. Acceleration Transfer Coefficient

To understand the propagation of ground motion from the soil layer to the structure, the ratio of the acceleration response of the structure to that of the soil layer is defined as the acceleration transfer coefficient *β* from the soil layer to the structure, which can be expressed as:

(1) $$\beta =\frac{a_{2}\;}{a_{1}}$$

In the formula, $a_{1}$ represents the peak acceleration near the structural foundation in the soil layer, and $a_{2}$ represents the peak acceleration at the structural foundation.

**Figure 17:**
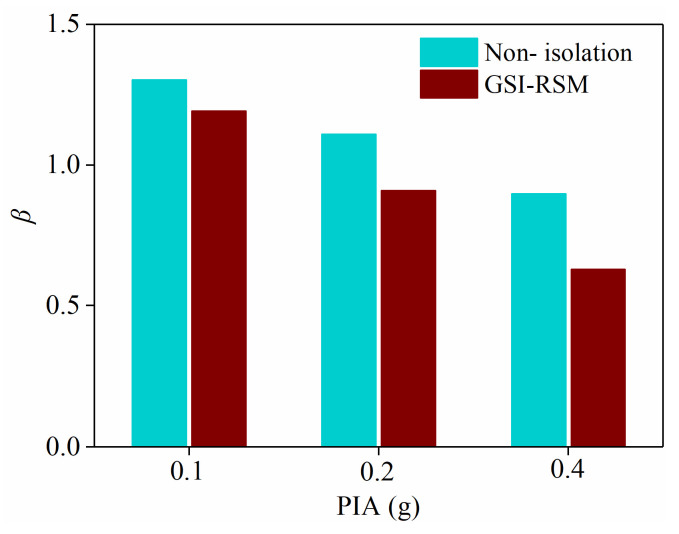
Acceleration response transfer coefficient.

The transfer coefficient of the acceleration response from the soil layer to the structure is shown in Figure 17, in which the acceleration response recorded at measuring points SA1 and A8 in the structure and soil layer were, selected respectively. It can be seen that when the input acceleration amplitude was 0.1 g, the acceleration transfer coefficient of the GSI–RSM test and the non-isolated test was the same. When the input acceleration amplitude was 0.2 g and 0.4 g, the transfer coefficient of the acceleration response from the soil layer to the structure decreased gradually, and the transfer coefficient of the GSI–RSM test was 13.3% and 26.6% lower than that of the non-isolation test, respectively., which shows that the GSI–RSM system limited the transfer of seismic motion to the upper structure and reduced the seismic input of the structural basement. Combined with the analysis of experimental phenomena, RSM had the characteristics of low modulus and large damping. In the GSI–RSM test, RSM filtered part of the high-frequency components of the seismic transmission to the upper structure and produced friction slip in the interaction with the structural foundation, which consumed more seismic energy and reduced the transmission of seismic energy to the upper structure.

#### 4.2.3. Structural Acceleration Response

**Figure 18:**
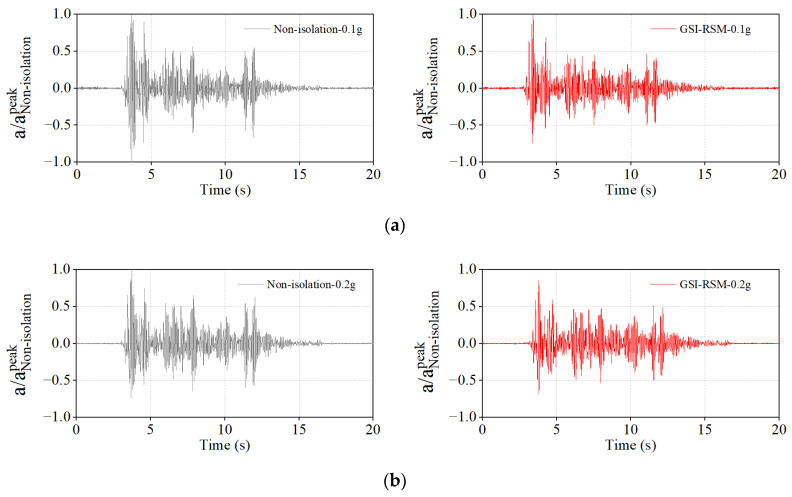

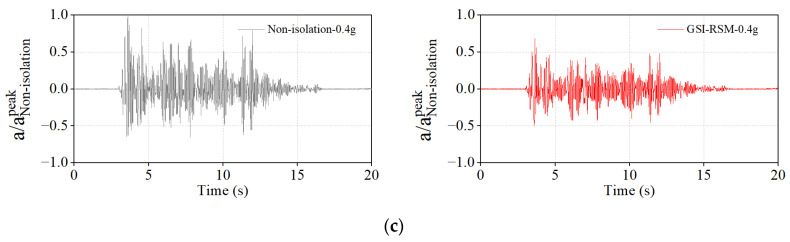
Normalized acceleration time–history curve of structural roof: (**a**) input acceleration amplitude of 0.1 g; (**b**) input acceleration amplitude of 0.2 g; (**c**) input acceleration amplitude of 0.4 g.

The acceleration time–history curve of the structural roof panel is shown in Figure 18; here, the acceleration was the normalized acceleration value of the peak acceleration through the non-isolation test. It can be seen from the diagram that when the input acceleration amplitude was 0.1 g, the peak acceleration response of the structural roof panel in the GSI–RSM and non-isolation tests was the same. When the input acceleration amplitudes were 0.2 g and 0.4 g, the peak accelerations of the structural roof panel in the GSI–RSM test were only 85.1% and 68.7% of those in the non-isolation test; that is, the peak accelerations of the structural roof panel were reduced by 14.9% and 31.3%, respectively. With the increase of the input acceleration amplitude, the reduction of the acceleration response of the GSI–RSM system to the structural roof panel was also gradually increased.

### 4.3. Displacement Responses

#### 4.3.1. Relative Displacement Response

The relative displacement time–history curve between the structural foundation and the RSM layer represents strong evidence to determine whether a slip has occurred between the structure and the RSM layer. At the same time, the maximum slip and the residual slip during the slip process can be seen through the time–history curve. The residual slip can show the re-centering ability of the GSI–RSM system, which determines the usability of the structure after the event.

**Figure 19:**
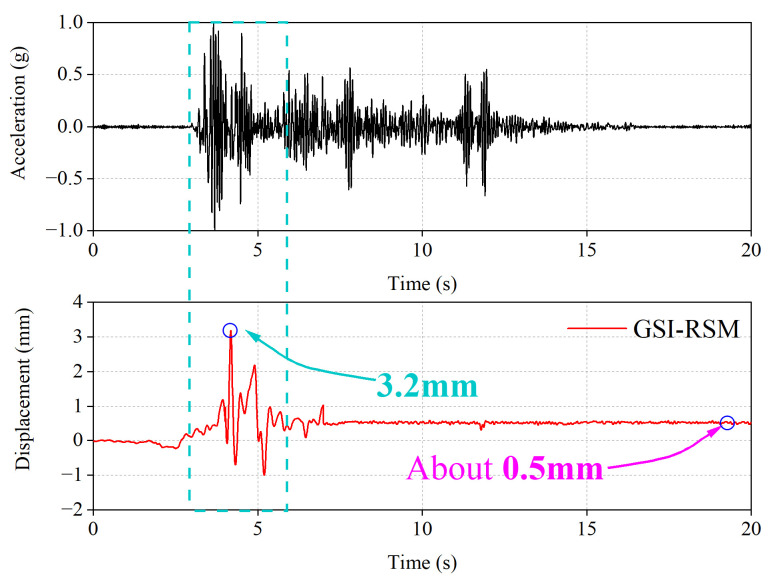
Relative displacement time–history curve.

Figure 19 shows the relative displacement time–history curve between the structural foundation and the RSM layer in the GSI–RSM test when the input acceleration amplitude was 0.4 g. It is clear that in the GSI–RSM test, the relative slip motion occurred between the structural foundation and the RSM layer, the relative slip displacement peak was 3.2 mm, and the relative slip displacement time was consistent with the maximum acceleration amplitude time range. It is worth noting that the residual slip displacement between the structural foundation and the RSM layer was only 0.5 mm. The residual displacement is just what should be at the end of the ground motion and is beneficial to the usability of the structure after the event.

#### 4.3.2. Storey Drift of the Structure

The storey drift of a structure is an important parameter to evaluate the deformation state of structures. The peak displacement and storey drift of structures is shown in Table 5. It can be seen from the table that when the input acceleration amplitudes were 0.2 g and 0.4 g, compared with the non-isolation test, the storey drift of the structure in the GSI–RSM test was significantly reduced, and the reduction rates of the storey drift reached 17% and 36%, respectively. When the input acceleration amplitude was 0.1 g, the storey drift of the two groups of tests had little difference. It can be seen that the damping effect of the GSI–RSM system was small when the magnitude of the earthquake was small, while the damping effect was good when the magnitude of the earthquake was large, which is consistent with the research conclusions of existing scholars on the influence of ground motion amplitude on the isolation effect of the GSI–RSM system [16].

## 5. Conclusions

The GSI–RSM system has the advantages of low cost and simple construction, and it concurrently uses waste tires as rubber particle materials. Therefore, it is a cheap and environmentally friendly isolation technology, which is very appropriate for promotion in rural residential buildings. However, to apply this technology to practical projects, more research need to be done in large-scale laboratory and field tests. Therefore, in this study, large shaking table tests were carried out on a 1/4 scale masonry model with and without a GSI–RSM system, and the following conclusions were obtained:

(1) The shaking table test results showed that the isolation effect of the GSI–RSM system was limited in small earthquakes, and the isolation effect of the GSI–RSM system increased with increasing earthquake magnitude. When the input acceleration amplitude was 0.4 g, the GSI–RSM system could reduce the acceleration response of the roof panel and the interlayer displacement response of the structure by more than 30%, which greatly reduced the seismic demand of the superstructure, achieved the purpose of isolation, and verified its effectiveness.
(2) Through the analysis of the shaking table test results, the isolation mechanism of the GSI–RSM system was mainly reflected, given that the RSM layer could filter part of the high-frequency components of the earthquake to transmit to the superstructure, and the RSM layer generated friction slip in the interaction with the structural foundation, which consumed more seismic energy and thus greatly reduced the seismic energy transmitted to the superstructure.
(3) When the input ground motion amplitude was 0.4 g, that is, the ground motion intensity was large, the relative slip movement between the structural foundation and the RSM layer occurred in the GSI–RSM test. The residual slip displacement between the structure foundation and RSM layer was only 0.5 mm, which is exactly the displacement at the end of the ground movement and is conducive to the availability of the structure after the event.

## Figures and Tables

**Table 1:** Similarity ratios of the structural model.

Physical Quantity	Similarity Relation	Similarity Ratio	Physical Quantity	Similarity Relation	Similarity Ratio
Length	*S_l_*	0.25	Density	*Sρ*	2.00
Elastic Modulus	*SE*	1.00	Time	*S_l_(S_ρ_*/*S_E_) ^0.5^*	0.35
Strain	*Sσ*/*SE*	1.00	Frequency	*(S_E_*/*S_ρ_) ^0.5^*/*S_l_*	2.83
Stress	*Sσ = SE*	1.00	Acceleration	*S_E_*/*(S_l_S_ρ_)*	2.00

**Table 2:** Loading sequence.

Condition	Seismic Wave	Direction	Input Acceleration Amplitude [g]
1	EL Centro wave (NS)	Horizontal	0.1
2	EL Centro wave (NS)	Horizontal	0.2
3	EL Centro wave (NS)	Horizontal	0.4

**Table 3:** Test data of masonry specimens.

Specimen Number	Sample Bottom Area (mm^2^)	Failure Load (kN)	Compressive Strength (MPa)	$\mathrm{Axial}\;\mathrm{Strain}\;\mathrm{at}\;0.4\;\mathrm{Times}\;f_{c}\;(\mu \varepsilon )$	Elastic Modulus (MPa)	Average Modulus of Elasticity (MPa)
1	15,480	51.3	3.32	1352	982.25	1031.97
2	15,130	46.7	3.09	1186	1042.16	
3	15,260	45.2	2.96	1105	1071.49	

*f_c_* is peak compressive stress.

**Table 4:** Basic physical and mechanical parameters of soil.

Type of Soil	Density (g/cm^3^)	Moisture Content (%)	Internal Friction Angle (°)	Cohesion (kPa)	Initial Shear Modulus (MPa)	Reference Strain
Natural soil	1.88	17.31	26.36	37.61	29.3	0.0297
RSM	1.48	6.02	22.87	5.43	18.5	0.0093

**Table 5:** Peak displacement and storey drift of structures.

Condition	Input Acceleration Amplitude of 0.1 g		Input Acceleration Amplitude of 0.2 g		Input Acceleration Amplitude of 0.4 g	
	Peak Displacement (mm)	Storey Drift (%)	Peak Displacement (mm)	Storey Drift (%)	Peak Displacement (mm)	Storey Drift (%)
Non-isolation	1.26	0.15	2.25	0.27	4.24	0.51
GSI–RSM	1.28	0.15	1.87	0.23	2.68	0.32

## Data Availability

Not applicable.
